# Soft Interaction in Liposome Nanocarriers for Therapeutic Drug Delivery

**DOI:** 10.3390/nano6070125

**Published:** 2016-06-25

**Authors:** Domenico Lombardo, Pietro Calandra, Davide Barreca, Salvatore Magazù, Mikhail A. Kiselev

**Affiliations:** 1 National Research Council, Institute for Chemical and Physical Processes, Messina 98158, Italy; 2 National Research Council, Institute of Nanostructured Materials, Roma 00015, Italy; pietro.calandra@ismn.cnr.it; 3 Department of Chemical Sciences, biological, pharmaceutical and environmental, University of Messina, Messina 98166, Italy; dbarreca@unime.it; 4 Department of Physics and Earth Sciences, University of Messina, Messina 98166, Italy; smagazu@unime.it; 5 Frank Laboratory of Neutron Physics, Joint Institute for Nuclear Research, Dubna, Moscow 141980, Russia; kiselev@jinr.ru

**Keywords:** liposomes, drug delivery, phospholipids vesicles systems, nanotechnology

## Abstract

The development of smart nanocarriers for the delivery of therapeutic drugs has experienced considerable expansion in recent decades, with the development of new medicines devoted to cancer treatment. In this respect a wide range of strategies can be developed by employing liposome nanocarriers with desired physico-chemical properties that, by exploiting a combination of a number of suitable soft interactions, can facilitate the transit through the biological barriers from the point of administration up to the site of drug action. As a result, the materials engineer has generated through the bottom up approach a variety of supramolecular nanocarriers for the encapsulation and controlled delivery of therapeutics which have revealed beneficial developments for stabilizing drug compounds, overcoming impediments to cellular and tissue uptake, and improving biodistribution of therapeutic compounds to target sites. Herein we present recent advances in liposome drug delivery by analyzing the main structural features of liposome nanocarriers which strongly influence their interaction in solution. More specifically, we will focus on the analysis of the relevant soft interactions involved in drug delivery processes which are responsible of main behaviour of soft nanocarriers in complex physiological fluids. Investigation of the interaction between liposomes at the molecular level can be considered an important platform for the modeling of the molecular recognition processes occurring between cells. Some relevant strategies to overcome the biological barriers during the drug delivery of the nanocarriers are presented which outline the main structure-properties relationships as well as their advantages (and drawbacks) in therapeutic and biomedical applications.

## 1. Introduction

Liposomes composed of surfactants, natural or synthetic (phospho)lipids, and block copolymer vesicles represent a versatile platform for the development of improved drug delivery therapies in a wide range of biomedical applications [1]. The ability of liposomal systems to entrap both lipophilic and hydrophilic compounds enables a large variety of drugs to be encapsulated by these vesicles, including deoxyribonucleic acids (DNA), proteins, or imaging agents [2,3]. The use of liposomes based nanocarriers in drug delivery technologies offers many benefits connected with their capacity for a versatile self-assembly, governed by a number of soft interactions that regulate colloidal stability of therapeutic drugs in bio-environments, thus preventing unwanted tissue and cellular uptake [4,5,6,7,8]. Liposome nanocarriers are characterized, in fact, by easily controllable properties (such as particle dimension, morphology, charge, lipid composition, and functionalization of the surface with polymers and ligands) that are able to govern their colloidal stability both in vitro and in vivo. Despite the success of liposomal formulations in vivo and the continuous and progressive achievements in biomedical applications, a clear understanding of the bio-membrane interaction is still far to be attained.

Herein, we analyze how the efficacy of liposomes depends on their physicochemical properties as well as on the nature of their components, on their size, lipid organization, and surface interactions (electrostatic, hydrogen bond, Van der Vaals, steric repulsion). In addition, we aim to highlight the main liposome properties that strongly influence their colloidal stability during their employment in drug delivery applications and their interaction with cells. We also describe some strategies developed to overcome the limitations of the biological barriers, and how those approaches have been crucial for the development of advanced smart drug delivery approaches.

## 2. Basic Concepts of Liposomal Nanocarriers

Liposomes and vesicle-like nanocarriers are hollow spherical structures formed by the self-assembly of surfactants and natural and/or synthetic lipids or block copolymers in aqueous solution. Due to their loading capacity, good biocompatibility, and the facile inclusion of tissue-recognition ligands, liposomes are suitable soft nanocarriers for a wide number of therapeutic applications. Nanocarriers formed by the self-association of phospholipids are versatile prototypes for drug delivery applications [2,7]. Phospholipids are generally composed of one hydrophilic head and two hydrophobic tails. Depending on the composition of the phospholipids, liposomes can possess positive, negative, or neutral surfaces. When phospholipids are dispersed in water, they aggregate spontaneously into bilayers, which resemble the structures they form in biological membranes. The final organization, morphology, and physicochemical properties of lipids-based liposomes depend on the nature, size, and geometry of their lipid components, concentration, temperature, and surface charge [7,8,9,10,11].

According the elastic models of Helfrich [12] which describe the bending behavior of lipid bilayers’ bio-membranes, the curvature energy of a bilayer in vesicles is higher than in the stacked multilamellar liquid crystalline phase (in excess water). Therefore, an energy cost is necessary to generate curvature that transforms an undisturbed membrane into vesicles. Lipid bilayer vesicles in aqueous solution are then metastable structures which strongly depend on the conditions of preparation (i.e.*,* stirring, sonication, extrusion, microfluidification, or electroformation) [13]. In such cases the produced vesicles may contain small unilamellar vesicles (SUVs < 100 nm), large unilamellar vesicles (LUVs 100–1000 nm), or giant unilamellar vesicles (GUVs > 1 μm). Finally multilamellar vesicles (MLVs) are composed of concentric bilayer surfaces in an onion-like structure (hydrated multilayers). The size of liposome nanocarriers employed for bio-medical applications ranges mainly between 50 and 500 nm [8]. The difference in the drug release rate strongly depends on the phospholipid bilayers that the active drug has to cross during the release process. Generally, MLVs with large diameters are formed more easily and thus have a greater entrapped volume than ULVs. Unilamellar liposomes exhibit a much faster drug release rate than MLVs with more lamellar bilayers.

## 3. Amphiphilic Soft Nanocarriers: Micelles, Vesicles, and Bilayers

**Figure 1:**
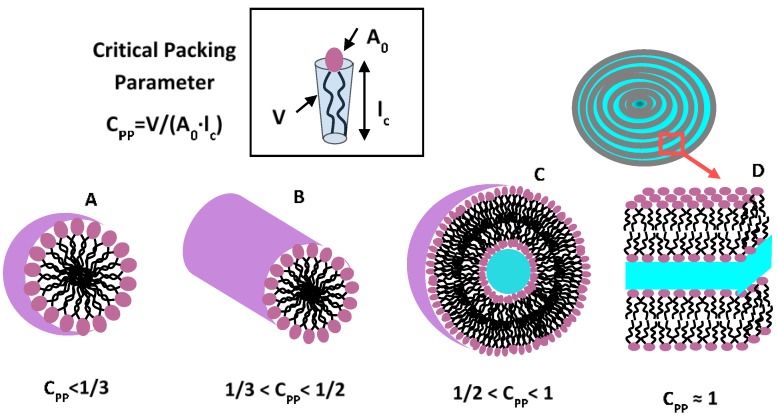
Relevant shape factor influencing nanocarrier morphology. Aggregate structures of amphilphilic molecules can be predicted from the critical packing parameter *Cpp*.

Amphiphilic macromolecules that form bilayer liposomes (vesicles) in aqueous solution possess both a hydrophilic and a lipophilic (or hydrophobic) part. The hydrophilic portion, which is called a “head group”, can be uncharged or charged (anionic, cationic, or zwitterionic) and interacts favourably with the surrounding water. The hydrophobic part (called a “tail”) is usually composed of hydrocarbon chains, and generally tends to minimize its exposure to water. In water solutions the hydration of the hydrophilic component, as well as the hydrophobic association of the tail(s), causes the formation of many micelle-like aggregates at a given concentration (critical micelle concentration, CMC) and temperature (critical micelle temperature, CMT) [6]. As the amphiphilic self-assembly is a dynamic process, the micellar aggregates present a dynamic structure in which the unimers are rapidly exchanged between micelles and the bulk solution (with lifetimes between 10^−5^ and 10^−3^ s) [6]. The shape and size of a given nanocarrier aggregate depends on the molecular geometry of its component surfactant molecules and the solution conditions, such as surfactant concentration, temperature, pH, and ionic strength [6,14]. Control over the shapes allows the possibility to develop and manipulate nanostructure architectures. According to Israelachvili et al. [6], a preliminary estimate of shape and size can be made with the analysis of the critical packing parameter *C_pp_* = *V*_0_/*A*_0_·*l_c_*, where *V*_0_ is the effective volume occupied in the aggregate core region by hydrophobic chains, *l_c_* is the maximum effective length (critical chain length), and *A*_0_ is the effective hydrophilic head group surface area at the aggregate-solution interface. As schematically reported in Figure 1, with the increment of the *C_pp_* value the structure of aggregates can range from spherical micelles (*C_pp_* ≤ 1/3), to cylindrical micelles (1/3 ≤ *C_pp_* ≤ 1/2), vesicles (1/2 ≤ *C_pp_* ≤ 1), and lamellar structures (*C_pp_* = 1) [6]. For larger values of the critical packing parameter *C_pp_* the amphiphiles will assemble into “inverted” phases [6,14].

## 4. Soft Interactions Involved in Liposome Nanocarriers

Interactions and forces between colloidal nanocarriers in suspensions play an important role in determining the properties of the materials, such as their stability, shelf life, and transport properties. Despite the weakness of the forces involved, the relevant number of these soft interactions will produce an overall effect that is strong enough to hold different molecular subunits together as well as ensure their stability in solution. Moreover, the weakness of the involved interactions makes the structure more flexible, thereby enabling the system to withstand minor perturbation while preserving the reversibility of the self-assembled structure. The main weak (non-covalent) forces acting on soft nanocarriers are hydrogen bonding, hydrophobic effects, electrostatic interactions, Van der Waals forces, and steric interactions. A summary of the strength and characteristic ranges of the main soft interactions are reported in Table 1.

### 4.1. Hydrogen Bonding and Hydrophobic Forces

Hydrogen bonds are fundamental interactions for many relevant functions in bio-systems, as they are strong enough to bind biomolecules together but weak enough to be broken inside living organisms and cells when necessary. For example, water hydrogen bonding has many important implications on the properties of water and its relevant functions in biological systems [15]. In water the nonbonding electrons are the H-bond acceptors and the hydrogen atoms are the H-bond donors. This polarity within water molecules results in an attraction force between the negative portion (of oxygen) and positive partial charges (of hydrogen). H-bond acceptors and donors in biomolecules are due to the presence of molecular dipoles of hydrogen’s atoms H (H-bond donors) bonded with electronegative atoms like oxygen O, nitrogen N, or fluorine F (H-bond acceptors). Hydrogen bonding is an important component of the major macromolecules in biochemistry (i.e., proteins, nucleic acids, and carbohydrates). The hydrogen bonding of proteins provides the organization for distinct folds and also provides the selectivity in protein-ligand interactions in molecular recognition processes. Apart from protein-ligand interactions, hydrogen bonds are implicated in many intermolecular interactions, including those involving protein-liposome, protein-protein, and protein-nucleic acid complexes [16]. Hydrogen bonds between complementary nucleotides favour the connection of two strands of a DNA helix together, where the complementary base pairs of guanine and cytosine (or adenine and thymine) are connected together by means of hydrogen bonding. Moreover, hydrogen bonding between phosphate groups on DNA nucleotides favours a helical structure conformation for two strands of DNA. Each base can also form hydrogen bonds with the external water mediated environment. Despite the softness/weakness of the (internal/external) hydrogen bond interactions, the presence of a large number of hydrogen bonds in biological macromolecules make them highly stable systems in different water environments. Another important interaction connected with the hydrogen bond is the hydration (or solvation) force [16,17,18]. The peculiar polarisation of water molecules near the hydrophilic solute interfaces sensitively alters the liquid structure with respect to the structure usually found in bulk water. [18,19].

The hydrophobic effect describes the unfavourable interaction of non-polar substances with water, and plays an important role in many soft nanocarrier systems as it regulates the tendency of non-polar (hydrophobic) molecules to self-aggregate [20]. When a hydrophobic compound is inserted in water the disruption of the H-bonding water network favourites a rearrangement of the water molecules around the non-polar molecules, with the creation of larger cavities to accommodate an assembly of non-polar (solute) molecules. In this case water molecules, distorted by the insertion of the hydrophobic component, will create new hydrogen bonds thus inducing an ice-like cage structure (called a *clathrate*) around the hydrophobic molecules. The hydrophobic interaction can be traced back to an effective (mutual) attractive hydrophobic force, between the surfaces of non-polar molecules in water. The effect of this interaction is formation of entropically more favourable aggregated structures generated to minimize the disruption of the water structure of water molecules migrating from the bulk water. Hydrophobic interactions play important roles in the intramolecular and intermolecular associations of biomolecules in various biological phenomena including protein folding and stabilization of macromolecules such as proteins and bio-membranes [16]. For example proteins, with both polar and non-polar side-chains, fold in water with the non-polar groups largely turned inward in order to avoid water [21,22].

### 4.2. Van der Waals Forces

The Van der Waals interaction is generated by permanent (or induced) dipoles within the nanocarriers that may result in net attractive forces between biological nano-components. This interaction is given by short-range repulsive forces and a long-range attractive London dispersion force generated by a temporary (short-lived) dipole induced by a polarisation of the electron distribution of an adjacent atom. For example, uncharged hydrophobic alkyl chains of phospholipids which have no permanent dipole are dominated by Van der Waals interactions which drive the packing of hydrophobic tails inside the bio-membrane bilayers [21].

### 4.3. Elctrostatic Interaction: Electrical Double Layer (EDL)

As the lipid bilayer represents the backbone structure of cell membranes, the functioning of cells is mainly related to electrostatic and electrochemical processes that occur at (or across) their lipid membranes. For example, liposomes’ surface electrostatic potential has been shown to have a strong influence on the binding constant and uptake of active compounds, such as hematoporphyrin (a charged photosensitizer in photodynamic therapy of tumours) [9]. As the binding of active ionic species strongly affects the aggregation behaviour of cells, electrostatic interaction in charged liposomes represents a fundamental force for the investigation of bio-membrane/ion interactions.

**Figure 2:**
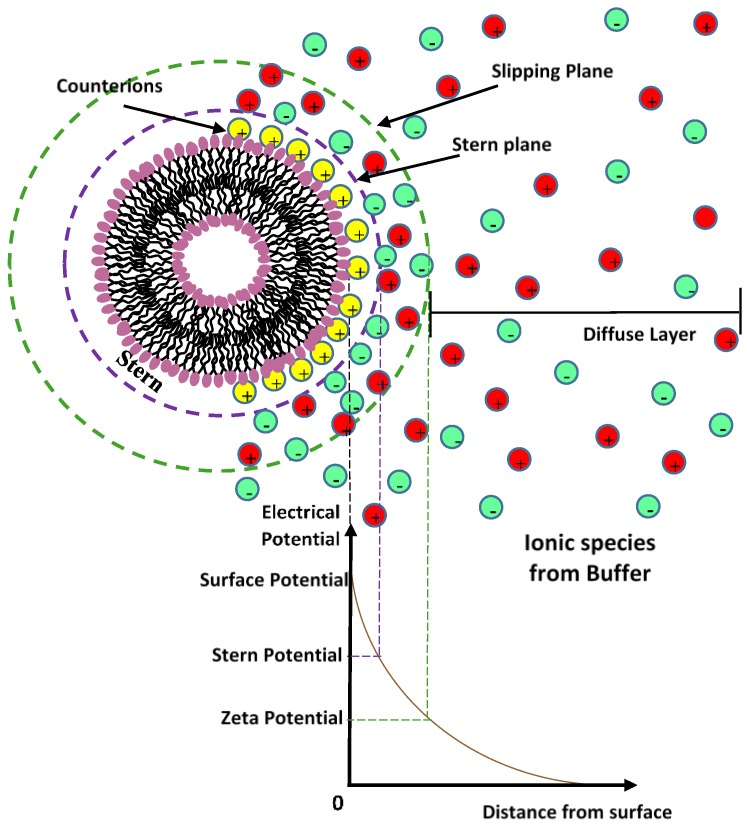
Schematic representation of the charge distribution around the charged surface of a liposome. The electrical double layer (EDL) is composed of a layer of ions strongly bound to the charged surface (Stern layer) and an adjacent region of loosely associated mobile ions (diffuse layer).

Most liposome nanocarriers carry some surface charge in aqueous environments due to the ionization/dissociation of surface groups, or adsorption of charged molecules/ions to the nanocarrier surface. The nanocarrier surface charge is balanced by a cloud of counterions around the particle (Figure 2). This cloud of charges, called an electrical double layer (EDL), is composed of a(n) (immobile) layer of ions strongly bound to the charged surface (Stern layer) and an adjacent region of loosely associated mobile ions (diffuse layer) [22]. Overlapping EDLs of two like-nanocarriers induces a repulsive force caused by the osmotic interactions between counterions. In a first approximation the thickness of the diffuse electric double layer is represented as the inverse of the Debye-Huckel screening constant κ which is determined, at a given temperature *T*, by the ionic strength *I* of the solvent (in mol/L) (where *e* is the unit of electron charge, *K_B_* the Boltzmann constant, and *N_a_* the Avogadro number) [22].

(1) $$\kappa =\sqrt{\frac{8\pi e^{2}N_{a}I}{\varepsilon K_{B}T\cdot 1000}}$$

The delivery mechanism and liposome efficiency in drug delivery processes are strongly influenced by the typology and density of the charge of the liposome surface through its ζ-potential [14,22]. A high electrostatic repulsion on the surface of charged liposomes prevents their aggregation and flocculation and could promote their interaction with cells [7,8,9,10,11,22]. Characterization of nanoparticle surface charge is usually done by measuring the particle zeta potential, which corresponds to the electrostatic potential of the nanocarriers measured at the so-called shear plane, (i.e.*,* from the surface where ions are not bound to the particle) [22].

### 4.4. Steric Stabilization Forces

Nanocarriers in a biological environment very often undergo aggregation in specific solution conditions [11,22]. Nanocarrier stability can be enhanced through the addition of small amounts of polymer to the solution medium. Both synthetic polymers and biopolymers are widely used as additives against aggregation, thus leading to the steric stabilisation of a nanocarrier in a wide range of solvents conditions. In general, the magnitude of the force between (polymers coated) surfaces depends on whether the polymer is simply adsorbed or irreversibly grafted (*stealth liposomes*) onto the nanocarrier surface, on the coverage regime of polymer (grafting regime on each surface), and finally, on the solvent quality (poor or theta solvent conditions) and charging regime (ionic strength) [23]. Due to the different conditions of specific biological systems encountered, steric interaction is not straightforward to describe. The force law between two flat surfaces covered with so-called polymer brushes can be derived by theoretical approaches based on polymer-scaling concepts. More specifically, the steric repulsive interaction (as a function of the distance *h*) between two liposomes with polyethylene glycol (PEG) polymer layers of thickness *L_brush_* and radius *R* can be expressed through an exponentially decaying force law, according to the following equation [23]:

(2) $$W{\left(h\right)}_{Steric}=\frac{100RL_{brush}^{2}}{\pi s^{3}}k_{B}T\exp\left(-\frac{\pi h}{L_{brush}}\right)$$

for 0.4 *L_brush_* < *h* < 1.8 *L_brush_*, where *L_brush_* = (*N_EO_*·*l*^5/3^/*s*^2/3^). *N_EO_* is the number of monomers (–OCH_2_CH_2_–) in the PEG chain, *l* is the effective segment length, and *s* is the distance between the grafting points [23].

## 5. Structural Characterization of Liposome Nanocarriers

The biological behaviour of liposomes is strongly influenced by control parameters such as shape, size, morphology, surface characteristics, and lamellarity. Those features play a crucial role [8,14] as they influence both their biodistribution and passive targeting abilities upon drug administration. For these reasons, liposome size, colloidal stability, and aggregation properties (as well as their time evolution) should be probed (in situ) in solution conditions that reproduce standard administration conditions. Among the experimental methods used, the small-angle neutron scattering (SANS) and small angle X-rays scattering (SAXS) are the most important and widely utilized non-destructive experimental approaches for the structural investigation of liposome-based complex nanocarriers [24,25]. More specifically, the formation of different structures can be efficiently evidenced by scattering experiments, thus highlighting the important role of relevant molecular conformations in many different processes of drug delivery approach. Scattering methods provide the basic items to examine the structure and interactions of nanoparticles in solution and to study the growth process and conformational changes that are relevant for the understanding (and prediction) of functional processes in bio-nanotechnology [26,27]. Electron microscopy techniques, such as scanning electron microscope (SEM) and cryo-TEM (transmission electron microscope) (or TEM using freeze-fracturing approach) provide an accurate determination of liposome size, since they allow for the visualization of individual liposomes and can resolve particles of varying size, morphology, and lamellarity [28,29]. Another important microscopic technique utilized to analyze liposome morphology and size is the atomic force microscopy (AFM); a technique which provides high resolution information on the three-dimensional profile of liposomes without the necessity of removing them from their native solution environment [29]. Different techniques are available for the determination of size and size distribution of liposome nanocarriers, among which the most widely applied include dynamic light scattering and size-exclusion chromatography. In dynamic (or quasielestic) light scattering (DLS or QELS, respectively) measurements of the laser scattered intensity-intensity time correlation function *g*_2_(*t*) allow information to be obtained about the hydrodynamic radius *R_H_* of diffusing nanoparticles in solution from the analysis of translational diffusion coefficient *D* = *K_B_T*/6πη*R_H_* (where *k_B_* is the Boltzmann constant, *T* the absolute temperature, and η the viscosity of the solvent) [30]. Detailed information about the profile of the liposome population over the whole range of sizes is also acquired by means of the size-exclusion chromatography technique (SEC) [31]. This technique is able to separate and quantify liposome populations by exploiting the time-based resolution of hydrodynamic size, where the separation process is based on the elution velocity from a column equipped with a porous substrate. Alternatively, by means of the *field-flow fractionation* (FFF) separation techniques, a field (thermal-gradient, centrifugal, electrical, or magnetic) is applied perpendicularly to the flow of a fluid colloidal suspension pumped through a narrow channel, which causes the separation of the particles within the fluid, depending on their differing mobilities under the force field exerted. Finally, Zeta (ζ) potential measurements can be useful to address the electrostatic effects in charged nanocarriers by measuring the electrophoretic mobility μ*E* using principles of phase analysis light scattering (PALS) [22,32]. By means of the measurement of the migration rate (i.e., velocity *v*) of charged nanoparticles in solution under the effect of an electric field *E*, an estimation of the electrophoretic mobility μ*E* of the particles (i.e., μ*E = v/E*) can be obtained, which allows for the calculation of the particles’ zeta potential, ζ, and the nanocarriers’ surface charge [32].

## 6. Passive Targeting in Nanocarrier Drug Delivery: Beyond the Biological Barriers

Passive targeting (i.e., when no specific targeting ligands are used) represents the major mechanism for many intravenously administered long-circulating liposome nanocarriers that satisfy the size constraints (i.e., 100–200 nm in diameter) and consequently are able to extravasate from vessels and translocate in tumour tissues, thus concentrating into the target site. Tumour (or inflammatory) tissues are characterized by increased vascular permeability. This effect is associated with malignant tissues that develop a discontinuous endothelium with large vascular fenestrations (pores) that allow liposomes of less than about 500 nm in size to diffuse outside the blood vessels (extravasation), thus entering the tumour interstitial space. The presence of pores (with diameters between 100 and 500 nm) induces extravasation of most drug-loaded liposomes of small dimensions [33,34]. After their administration, liposome nanocarriers are then sequestered from the circulation through fenestrations in their microvasculature, and will accumulate in the organs of the *mononuclear phagocyte system* (MPS) (also known as *reticulo endothelial system*, RES). Although the liver and the spleen exhibit the largest capacity for liposomal uptake (as they present sensitive blood irroration), liposome nanocarriers will also accumulate in minor quantity in the lungs, kidney, bone marrow, and lymph nodes. Liposomes undergo successive clearing in the MPS by resident macrophages via direct interactions with the phagocytic cells [33,34,35]. In addition, the interaction of liposomes with blood proteins plays a crucial role in the clearance process and tissue distribution of intravenously injected liposomes. During their circulation in the bloodstream, liposomes interact with plasma proteins, such as opsonins, high-density lipoproteins (HDLs), and low-density lipoproteins (LDLs) [36]. Opsonins include various protein types (such as immunoglobulin, fibronectin, lipoproteins) which help MPS to recognize and eliminate liposomes through the vesicle opsonization process, consisting of the adsorption of plasma proteins onto the phospholipid membrane. Elimination of liposomes takes place in different ways. A first way involves absorption of plasma proteins on the liposome surface followed by their recognition by the MPS. This results in the excretion of the drug cargo at the hepatic level and its successive metabolism by Kupffer cells. In a second way, liposomes are metabolized by the splenic macrophages. Finally, after their accumulation, liposomes are metabolized and eliminated by the target tissues. The clearance process by the MPS via serum proteins’ opsonization depends on a variety of factors including size, surface charge, and colloidal stability [37]. Generally, large negatively charged liposomes are eliminated more rapidly than small, neutral or positively charged liposomes [37]. Concerning the high-density lipoproteins (HDLs) and low-density lipoproteins (LDLs) contained in the blood, they interact with liposomes giving rise to a reduction of their colloidal stability. More specifically, the interaction with lipoproteins causes lipid transfers (and consequent lipid depletion) and structural changes on the surface of liposomes, followed by the liposome destruction and rapid release of the cargo to the plasma [36,37]. Interaction of liposomes with key blood proteins—such as immunoglobulins, complement proteins, apolipoproteins, fetuin, and thrombospondin—has been shown to play an important role in liposome recognition by specific phagocytes and (non-macrophage) hepatic cells [8]. In this case, the binding of blood proteins to liposomes strongly depends on the vesicles’ properties, such as their morphology, surface curvature and charge, lipid bilayer composition, and packing [8]. Alternatively, non-opsonic blood proteins can be adsorbed onto particle surfaces and experience conformational changes that probably expose chemical groups that could either be recognized directly by certain phagocyte cell surface receptors or could act as ligands for subsequent recognition by blood opsonins. A number of specific plasma membrane receptors (such as lectin receptors, CD14 receptor), or various classes of scavenger receptors (e.g., classes A, B, and D), has been identified in this specific phospholipid recognition process [8].

## 7. Drug Delivery Mechanism

When a liposome interacts with a cell, the delivery of the drug and its distribution in the target cell can occur in several ways [33,34,35,36,37]. A first mechanism consists of liposome’s adsorption into the cell membrane, where the lipid bilayer of the nanocarrier is degraded by mechanical strain or the action of enzymes. The active drugs will be released into the extracellular fluid, where they will diffuse through the cell membrane and the cytoplasm. A second way consists of the fusion of the liposomal membrane with the plasma membrane of the target cell, with the release of liposomal content directly into the cytoplasm [38]. Finally, the third way is the receptor-mediated endocytosis [39]. This frequent process only regards vesicles with a maximum diameter of 150 nm and the active ingredients that can endure the acidic environment of lysosomes (where liposomes undergo the enzymes’ action) [40]. Phagocytosis can also occur, but involves liposomes larger than 150 nm that are phagocytosed by specialized cells of the immune system, (such as macrophages, monocytes, and Kupffer cells) [33,34,35,36,37].

## 8. Strategies to Prolong the Circulation Time

When injected into blood circulation liposomes rapidly interact with the biological system. The clearance of circulating liposomes nanocarriers from the bloodstream, coupled with their high uptake by the MPS, represent an obstacle to any attempt at targeting to tumours. In order to preserve the optimal liposome nanocarrier efficiency, the organism’s defenses must be circumvented by avoiding the liposome nanocarrier recognition and the consequent neutralization and elimination of the invading active drugs. The physico-chemical properties of nanocarriers, such as size (and morphology) and surface functionality (charge typology, stiffness), are the main parameters that can affect their biological clearance. Different strategies can be adopted to prolong the circulation time, while the relevant mechanisms of stabilization can be exploited to improve colloidal stability in biological media [41,42].

A first approach to prolong the release rate of entrapped active substances consists of the choice of drugs with enhanced hydrophobic character. This characteristic promotes a slow release and prolongation in the liposome nanocarrier for up to several days. Moreover, prolongation in the circulation time (of several hours) in neutral liposomes has been obtained by incorporating cholesterol lipids [41,42]. Due to its hydrophobic character, cholesterol preferentially interacts with the core region of the membrane, thus inducing a dense packing of phospholipids. This reduces their permeability (*bilayer-tightening effect*) and increases their in vivo and in vitro stability, thus inhibiting their transfer to high-density lipoproteins (HDLs) and low-density lipoproteins (LDLs). Favourable results were also obtained with liposomes containing a small molar fraction of a negatively charged glycolipid, such as phosphatidylinositol. The bio-distribution of such nanocarriers evidenced therapeutic potential in cancer treatment for increasing the concentration of cytotoxic agents in tumour tissues while minimizing the likelihood of toxicity to the MPS [42].

### 8.1. Electrostatic Stabilization 

One approach to stabilize liposome nanocarriers in solution consists of the inclusion of electrostatic repulsive forces by including charged components that confer a net (positive or negative) charge. Charged liposome have many advantages compared with neutral ones. For example, the presence of a surface charge induces electrostatic repulsion among liposomes by creating a sensitive ζ-potential that prevents aggregation or flocculation processes in solution. Moreover, a high surface charge could promote the interaction of liposomes with the cells [43]. For example, it has been shown that modification of the nanocarrier surface charge can influence the electrostatic interaction of the nanocarriers with components in the gastrointestinal tract following oral administration, thus conferring selectivity to diseased tissue [44]. Some investigations indicate that negatively charged liposomes are less stable than neutral and positively charged liposomes when injected into blood circulation, as they rapidly interact with the biological system following their opsonization with circulating proteins, thus inducing toxic effects and a rapid uptake by the MPS [45]. 

On the contrary, cationic liposomes are mainly preferred for gene delivery, based on the electrostatic interaction between phospholipids such as the lipid dioleoylphosphatidylethanolamine (DOPE) which have a positive charge from the amine head group (NH^3+^) and negatively charged nucleic acids [46]. Generally, neutral phospholipids are, in most cases, also required for the stabilization of the liposome/DNA complex [47,48]. Moreover, they showed enhanced anti-tumour efficiency when associated with anti-tumoural drugs, such as Doxorubicin [49]. Cationic liposomes may also favour the interactions with the glycoproteins of the endothelial cell membranes [50] and the achievement of longer circulation half-lives [51]. Together with the number of advantages provided by the employment of cationic nanocarriers, some drawbacks should also be pointed out. The enhancement of the positive charge may favour, in fact, cationic liposome aggregation with anionic species present in the blood, thus inducing an unwanted enhanced uptake by the MPS and a diminished accumulation in tumour tissues [52]. Finally, it is worth noting that together with size- and coating-dependent uptake of nanocarriers by microvascular endothelial cells [53,54,55] that physical forces such as cyclic shear stress and cyclic stretch (under the physiological conditions of the pulsatile blood flow) may also affect cell-nanoparticle interactions resulting in reduced uptake into endothelial cells [56].

**Figure 3:**
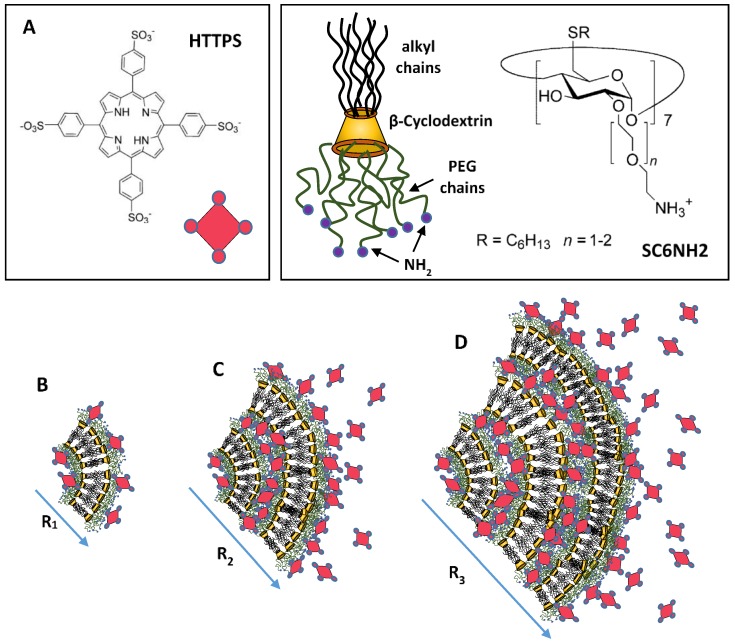
Models for the heterotopic aggregates formed by the self-assembly of anionic porphyrins (HTTPS) entangled in cationic amphiphilic modified cyclodextrins (SC6CDNH2) (**A**), at a different porphyrins/cyclodextrins (TPPS/SC6CDNH2) ratio. Reduction of the relative amount of modified cyclodextrin causes an increase in aggregate dimension as evidenced at the different ratio [TPPS/SC6CDNH2] = 1:50 (**B**), 1:2 (**C**), and 1:1 (**D**) [58].

It is worth pointing out that in some specific cases the supramolecular self-assembly processes combined with cooperative charge effects may enhance the stability to the nanocarrier during their heterotopic aggregate formation. Investigation of a novel heterotopic nanoparticle formed through the electrostatic interaction between the anionic porphyrin 5,10,15,20-tetrakis(4-sulfonatophenyl)-21H,23H-porphine (TPPS) entangled in cationic modified amphiphilic cyclodextrins indicated the important role of nanocarrier charges in the modulation of colloidal stability in solution [57]. As reported in Figure 3, the morphological evolution of the heterotopic nanoparticle formation indicates that sensitive structural rearrangements, essentially driven by electrostatic interaction, are responsible for a significant increase of vesicle dimensions connected with a charge equilibration within the system. Further investigation in anti-cancer drug delivery experiments evidenced that, in the range of porphyrin/CD molar ratios between 1:10 and 1:50, the porphyrin is solubilized in monomeric form and photosensitizes the production of singlet oxygen (^1^O_2_) for cancer therapy treatments [58]. Moreover, at the same molar ratio the ability of the investigated amphiphilic cyclodextrin to transport porphyrins into tumour cells indicated specificity at the nuclear compartment level [58]. These findings are of paramount importance for the development of a new generation of biocompatible cyclodextrin-based smart liposome nanocarriers for the photodynamic therapy of tumours. It is worth stressing that combined steric and electrostatic interaction generated by drugs inclusion may induce structural transitions in liposomes that strongly influence the structural stability of the nanocarrier, as demonstrated by many studies performed in bio-membranes [57,58,59,60,61,62].

### 8.2. Steric Stabilization

**Figure 4:**
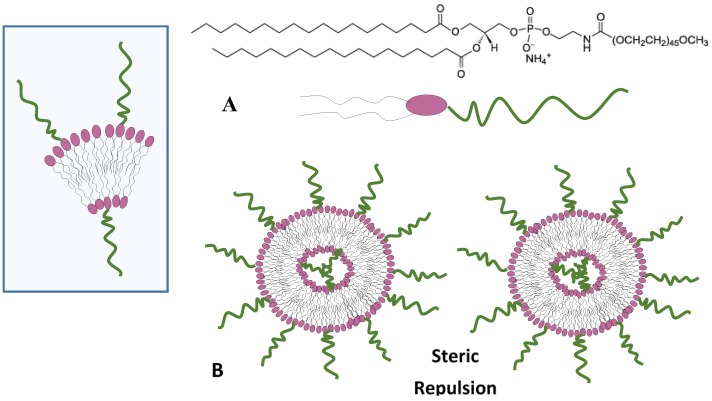
Schematic representation of the PEGylated phospholipid (DSPE-PEG2000) 1,2-distearoyl-sn-glycero-3-phosphoethanolamine-N-[methoxy(polyethyleneglycol)-2000] ammonium salt (**A**). View of sterically stabilised lipid bilayers (**B**). PEG, polyethylene glycol.

Another important approach for the improvement of circulation times consists of the conjugation of hydrophilic polymers to the surface of the liposome nanocarriers. This approach is generally accomplished by the conjugation on the nanocarrier surface of natural (e.g., dextran, alginate, chitosan) or synthetic (e.g., poly(ethylene glycol), PEG; poly(vinyl alcohol), PVA; poly(vinyl pyrrolidone), PVP) polymers [62]. This approach allows most of the challenges encountered by liposome nanocarriers in drug delivery processes (such as the low blood circulation half-life, toxicity, interception by the immune system, biocompatibility, and antigenicity issues) to be overcome. Among the hydrophilic polymers, PEG represents the most widely used polymer conjugate. PEGylation of the liposome surface creates a local surface concentration of highly hydrated polymer brushes (Figure 4). This hydration shell around the particle sterically inhibits both hydrophobic and electrostatic interactions with plasma proteins or cells, thus reducing the liposomal uptake process by the MPS [63]

As a result, PEGylated liposomes are not opsonized and are able to escape the capture by the cells’ phagocytic systems by rendering the nanocarriers invisible to macrophages (“Stealth liposomes”). Many studies demonstrated that PEGylated liposomes were able to improve the stability and blood-circulation time, together with low plasma clearance, and low volume of distribution (with minimal interaction with non-tumoural tissues) [64,65,66]. Chen et al. [67] showed that plasma protein adsorption onto nanocarrier surfaces on PEG-coated nanocarriers strongly depends on the PEG chain length and density at the nanocarrier surface. 

It is worth noting that the ability of PEG to increase the circulation lifetime of the vehicles strongly depends on both the amount of grafted PEG and the length or molecular weight of the polymer [64], while high molecular weight of entangled PEG chains may prefer aggregation in large clusters [68]. Interpenetration of long hydrophilic PEG chains belonging to different nanocarriers may cause, in fact, the depletion of the solvent in the outer layer of the nanocarrier, thus favouring the formation of larger clusters of entangled aggregates [69,70]. Temperature-dependent attractive interaction arising from the synergistic effects of different coexisting interactions in the system [71,72] can be exploited for triggering the assembly/disassembly process in PEG-based nano-aggregates. Interestingly, recent computer simulations and fluorescence experiments [73] evidenced that a model hydrophobic drug 5,10,15,20-tetrakis(4-hydroxyphenyl)porphyrin (*p*-THPP), besides being embedded deep within the hydrophobic core of the liposome, is also able to be wrapped by the hydrophilic PEG layer surrounding the lipid molecules, thus improving the drug loading efficiency of the liposome [73]. It is worth noting that the enhanced steric stability of PEG-bearing liposomes can have the drawback of a reduced ability to interact with the intended targets or not being able to completely avoid cumulative uptake by cells, as evidenced by a recent published review in this area [74].

## 9. Liposome-Liposome Interaction and Molecular Recognition Processes

Fusion and fission of vesicles are particularly important phenomena since they are at the basis of biological membrane processes such as vesicle–cell membrane fusion, endocytosis, exocytosis, and cell fission (cell division) [75,76]. Adhesion and fusion of biological membranes are mediated by non-covalent protein-protein and protein-carbohydrate interactions that may involve a range of receptor-mediated structural modifications involving tissue integrity and cellular rearrangements.

### 9.1. Liposome Aggregation-Fusion

Depending on the biological solution condition encountered, mutual liposome interaction can give rise to aggregation (or clustering), fusion, and fission processes [75,76]. In aggregation processes vesicles aggregate into large clusters, where each vesicle structure remains intact while its drug contents do not mix or leak out. On the contrary, in fusion processes vesicles merge into large vesicles, while their components mix, sometimes with leakage. Finally, in fission processes small vesicles are formed from large ones. Aggregation and fusion can be considered spontaneous processes occurring at slow rates and may eventually lead to flocculation and precipitation of the liposome. These interaction processes can also be induced by a number of external/internal factors (stimuli-responsive liposomes) [77,78,79]. Stimuli-responsive liposomes regulate the stability/permeability of the liposome nanocarrier by the inclusion of a triggerable component which has the task of reacting when triggered by stimuli from target tissues (pH, redox potential) or applied from outside the organism (light, temperature, hyperthermia, ultrasound, and (electro)magnetic field) [77]. For example, a pH-sensitive liposome which is stable at the physiological pH encountered in regular tissues will typically acquire fusogenic properties under the acidic environment generally encountered in pathological tissues, thus releasing its active drugs to nearby tumours and infected tissues.

### 9.2. Governing the Colloidal Stability in Bio-Systems

Experimental assessment of the colloidal stability in the biological environment is not a straightforward task and a wide range of different components further contributes to this complexity. When nanocarriers are delivered into cell culture media their properties are strongly influenced by the high ionic content that, very often, gives rise to an aggregation processes due to the attenuation of the screened electrostatic interactions and consequent suppression of the stabilizing effect of the electric double layer (EDL). Consequently, the destabilized suspension will rapidly collapse and the nanocarriers will aggregate.

A classical theoretical approach to determine the stability of a colloidal nanocarrier in solution is given by the Derjaguin–Landau–Verwey–Overbeek (DLVO) theory [78] in a pioneering work describing the stability of colloids in solution. According to this model, the interaction potential between two identical nanoparticles (i.e., spherical macroions of diameter σ = 2*R*) placed at a distance *r* (centre to centre distance) is determined by the balance between the Van der Waals attractive forces, *V_A_*, which is expressed as a function of the *A* which is the Hamaker constant (which depends on the dielectric constant mismatch between the particles and the surrounding fluid [22,78]).

(3) $$V_{A}=-\frac{A}{12}\left[\frac{1}{r^{2}-1}+\frac{1}{r^{2}}+2\ln\left(1-\frac{1}{r^{2}}\right)\right]$$

and the screened Coulomb (repulsive) interaction *V_R_* described by the following equation:

(4) $$V_{R}=\frac{{Z_{0}}^{2}e^{2}}{4\pi \varepsilon {\left(1+\kappa \sigma \right)}^{2}}\frac{e^{-\kappa (r-\sigma )}}{r}$$

**Figure 5:**
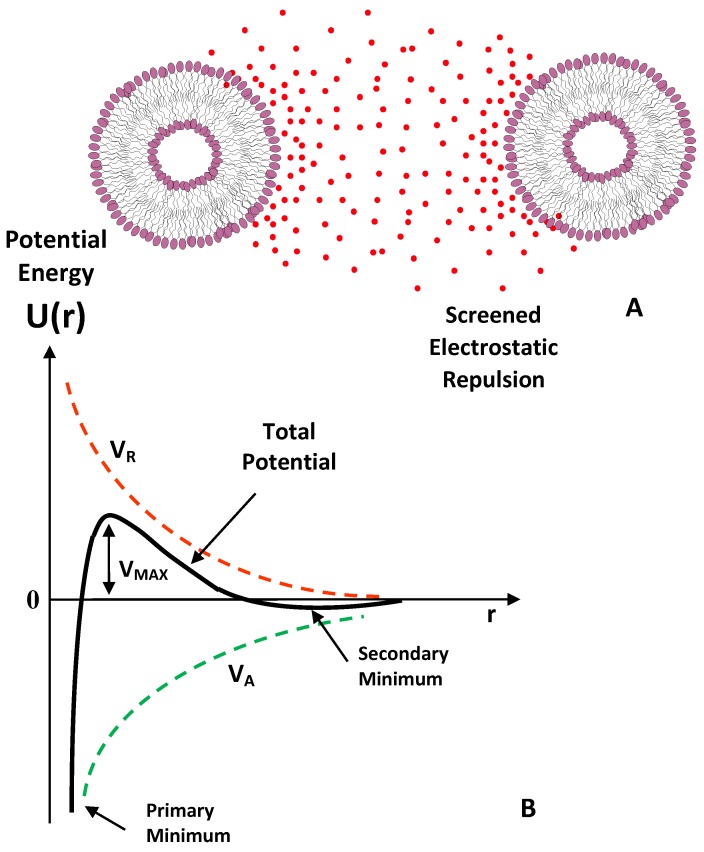
Sketch of the Derjaguin–Landau–Verwey–Overbeek (DLVO)-type interaction energy as a function of particle separation. The net energy is given by the sum of the double layer repulsion *V_R_* represented by a Yukawa type potential (electrostatic repulsion screened by ionic species in solution) and the Van der Waals attractive forces potential *V_A_*.

*e* is the unit of electron charge, *K_B_* the Botzmann constant, *N_a_* the Avogadro number, and *κ* is the Debye-Huckel constant (equation 1). A sketch of the DLVO interaction energy as a function of particle separation is reported in Figure 5. The presence of an energy barrier resulting from the repulsive force prevents two particles adhering together while approaching one another. Particles with a sufficiently high repulsion can avoid flocculation and the colloidal system will be stable. On the other hand, particles colliding with sufficient energy are able to overcome that barrier, thus favouring an aggregation process between particles. In certain solution conditions (e.g., at high ionic strength) there is a possibility of a “secondary minimum” where a weaker and reversible adhesion between particles may exist. These weak aggregates, although sufficiently stable against Brownian motion, may dissociate under an externally applied perturbation (i.e., due to a temperature increase).

As previously stated, the adsorption of synthetic polymers or biopolymers onto the nanocarrier’s surface can lead to a steric stabilization of the colloid that prevents two particles from forming attractive Van der Vaals interactions. It is worth pointing out that a Yukawa type potential is an adequate mathematical form to also properly describe the steric repulsion of polymer grafted nanocarriers. Moreover, the total interaction potential between nanocarriers in solution may be approximated by using two separate Yukawa potentials accounting for both the attraction and repulsion present in the system. This approach has been recently used to model, by small-angle neutron scattering (SANS) experiments, both the attractive and repulsive parts of the interaction between nanoparticles [79]. 

Despite the important information provided, the application of the DLVO theory for the analysis of the interactions in therapeutic nanocarrier systems in solution has the difficulty associated with having to rationalize the ionic strength parameters in the presence of charged components from a buffer solution. This is partially attributed to the difficulty of the theory to correctly describe the behaviour of the interface between particles and media, but also to the intrinsic experimental inaccuracy in the values of parameters, such as surface charge density, ligand density, and adsorption processes commonly encountered in drug delivery processes. SAXS and SANS structure factor S(q) analysis performed in the framework of liquid state theory [80] has been employed to investigate the interparticle interactions in different colloidal systems, including micelles, liposomes, dendrimers and protein [81,82,83,84].

### 9.3. Hydrogen Bonding Mediated Interactions between Liposomes and Molecular Recognition Processes

Interactions between liposomes at the molecular level through recognition processes outline strong similarities to the analogous cells incorporating recognizable amphiphiles in bio-membranes, and thus represent an important strategy for modeling the molecular recognition processes occurring between cells [85].

**Figure 6:**
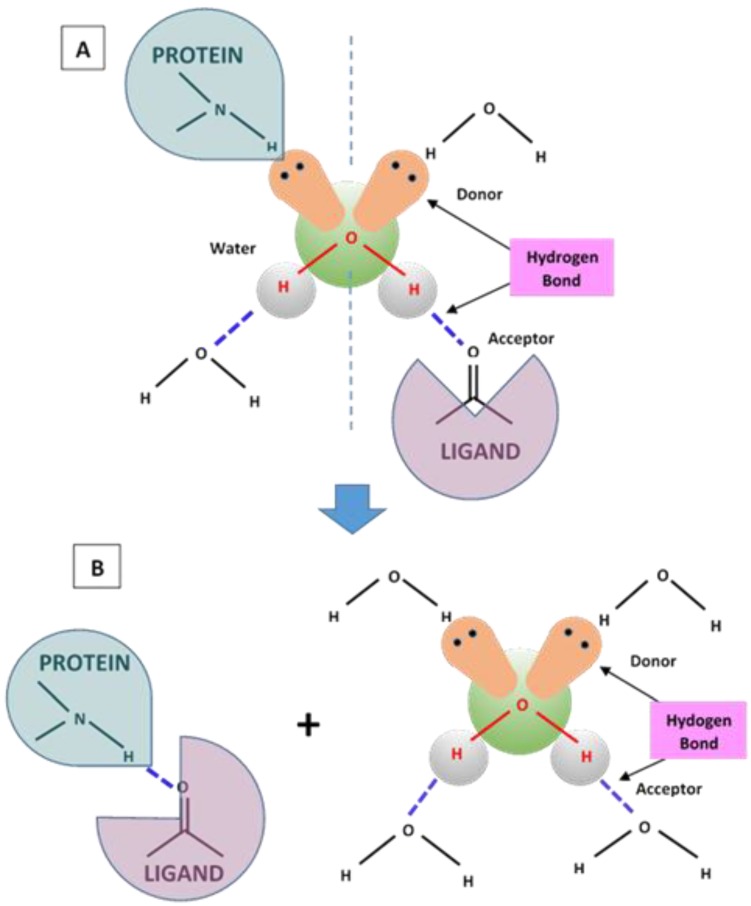
Hydrogen bond mechanism (solvation-desolvation) involved before (**A**) and after (**B**) the formation and structural organization of a ligand-protein complex.

**Figure 7:**
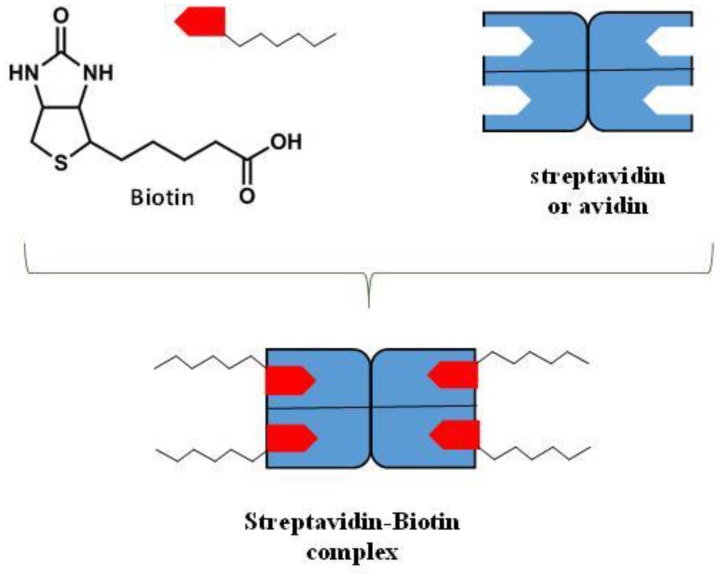
Schematic representation of the interaction of biotin with the tetrameric protein streptavidin.

Particularly interesting in this respect are the molecular recognition processes in bio-membranes through the hydrogen bonding interaction. Hydrogen bonding plays a key role in the molecular recognition of carbohydrates by membrane proteins. As an example, in the formation of ligand-protein complex, the binding of the ligand to its specific site will be favoured if the energy of the hydrogen bonds in the complex and the entropy gain in releasing some bound water molecules are more favourable than the free-energy contribution of the hydrogen bonds between the binding partners and these water molecules (in their free non-bonded state) (Figure 6). The introduction to liposome systems of hydrogen bonds forming functional groups can promote liposome bridging, thus leading to the formation of large aggregates. This process is sometimes followed by the liposome fusion in which the liposomes merge, and thereby share a common inner compartment. This second stage gives rise to the mixing of the aqueous content of liposomes with (or without) rupturing or leakage of the fused vesicles. It is worth noting that while vesicles aggregated by nonspecific forces, such as Van der Waals, are deformed and stressed, producing unstable structures, site-specific ligand-receptor coupling (such as biotin-streptavidin) promotes a secondary self-assembly into higher order structures composed of tethered (rather than adhering) vesicles in their original, unstressed state, as demonstrated by a Cryoelectron microscopy investigation [86]. Vesicle association by site-specific binding provides then an interesting mechanism for the production of easily controllable, stable, and reversible nano-biomaterials. As described by S Chiruvolu, et al. [86], hydrogen bonding can be used to aggregate vesicles into higher order structures in a reversible manner by means of site-specific ligand-receptor (biotin-streptavidin) coupling (Figure 7). In that case, biotin and streptavidin formed a very strong non-covalent complex (Ka ≈ 10^15^ M^−^^1^) by multiple tight hydrogen bonds.

**Figure 8:**
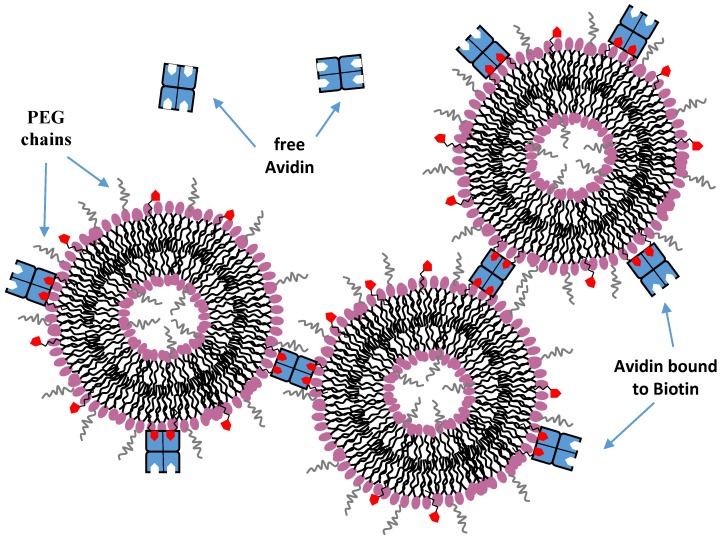
Representation of the aggregation of biotinylated liposomes induced by the tetrameric streptavidin protein.

Mixed liposome formed by PEGylated distearoylphosphatidylethanolamine lipid (PEG-DSPE) and biotin-(CH_2_)_n_-hexadecylphosphatidylethanolamine amphiphile showed the ability to form extended clusters of interconnected liposomes [87]. More specifically, avidin-coated liposomes interacted through biotin bridges leading to liposome-liposome adhesion. In this case, the insertion of fixed amounts of PEG (with molecular weight 750) linked at the head group of 1,2-Distearoyl-sn-glycero-3-phosphoethanolamine (DSPE) lipids created a repulsive steric barrier in order to accomplish the important function of eliminating non-specific liposome adhesion (Figure 8). It was found that the rate of avidin absorption decreased with the increasing amount of PEG750 introduced, while a 10 mol % PEG750 completely suppressed the binding of avidin to biotin. 

**Figure 9:**
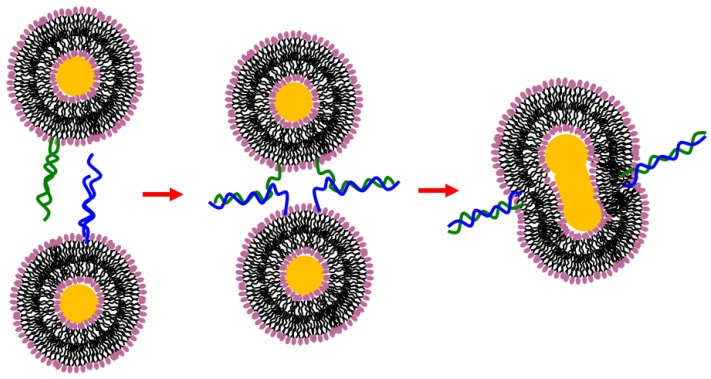
Schematic representation of the deoxyribonucleic acids (DNA)-induced vesicle fusion process [90].

An investigation evidenced how cyanuric acid (CA) and melamine (M) functionalized lipids promote the formation of bio-membranes that exhibit intense hydrogen-bond driven surface recognition in water, with variable hydration at the lipid-water interface. This system guided recognition at the lipid-water interface using cyanurate-melamine hydrogen bonding (incorporated at 0.1–5 mol percent) in fluid phospholipid membranes, and was able to induce both vesicle-vesicle binding (with multivalent surface clustering) and membrane fusion [88]. Further, an interesting example of multivalent and selective hydrogen bonding is provided by nucleic acids. For example, peptide nucleic acids (PNA) and deoxyribonucleic acids (DNA) may be anchored to liposomes by properly attaching hydrophobic blocks. This approach allows for the investigation of hybridization interactions with complementary strands of DNA by the binding of DNA to the PNA liposomes [89]. Stengel et al. reported fusion of liposomes induced by DNA hybridization [90], where some specific forced bilayer contacts are able to trigger vesicle fusion (Figure 9). This last example provides evidence once again for how receptors located at the surface of biological cells may facilitate adhesion or fusion with other cells. The prominent role of those functional groups, which render liposomes recognizable through hydrogen bonding, can be exploited to mimic analogous interactions occurring between cells. Morphological transition induced by molecular recognition is at the basis of intense investigations concerning protein-drug interactions and for understanding the mechanism behind versatile bioactivities of drugs [91,92,93,94].

## 10. Liposome-Drug Interaction: Encapsulation and Delivery Approaches

Liposomal nanocarriers are able to protect the drug cargos from undesired side effects or degradation before reaching target sites and reduce systemic toxicity of drugs and therapeutic agents. The delivery of liposome-encapsulated drugs depends on the structure and characteristics of the (host) liposomes, the size and nature of the (guest) drug molecules, and their mutual interactions. Many strategies can be adopted to improve both drug encapsulation and delivery efficiency to the target site in an effort to avoid undesired side effects. We will shortly review, in the following sections, the main strategies adopted to optimize the drug release with improved bioavailability in tumour tissues.

### 10.1. Hydrophilic/Hydrophobic Drug Encapsulation and Release Properties

The encapsulation of drugs into the bilayer of liposomes, while facilitating their solubilisation in aqueous media, also provides additional protection and control against drug degradation. These conditions implement a sensitive amelioration in the toxicity profiles with improved therapeutic efficacy with respect to the unencapsulated ones, as evidenced by several clinical studies [95,96,97]. Hydrophilic drugs are localized in the aqueous core region or near the liposome hydrophilic head groups (water–lipid interface) with an encapsulation efficiency that depends on the volume of the aqueous phase inserted during the liposome formation process. The encapsulation efficiency of hydrophobic drugs strongly depends on the length and packing density of the liposome acyl chains that host them, while an increased loading ability requires an increase in the bilayer lipophilic volume by choosing, for example, longer alkyl chain lipids [96,97]. Many anti-cancer drugs are of intermediate solubility, they undergo then a partition between the hydrophobic interior of the bilayer and the exterior (or interior) liposome aqueous phase. Liposome permeability plays a significant role in the encapsulation efficiency of hydrophobic/hydrophilic drugs, with the liquid crystalline phase that generally supports a more permeable character to the encapsulated drugs than the corresponding gel state [97]. The inclusion of macromolecules may also reduce lipid ordering giving a higher fluidity to the membrane and, in some cases, also inducing a local perturbation/disruption of the bilayer structure [98]. It is possible to switch from a fluid-phase to a solid-phase bilayer by incorporating cholesterol into liposomes (bilayer-tightening effect). The incorporation of cholesterol into liposomes stabilizes the lipid bilayer by reducing the rotational freedom (by increasing the orientation order) of the phospholipid hydrocarbon chains, thus inducing a more compact structure (with a reduced permeability) that causes an increased retention of the drug cargo in the liposomes.

### 10.2. Triggered Drug Release from Stimuli Responsive Liposomes (SRL)

Triggered drug release in a specific target tissue can be obtained with the inclusion of a triggerable component that allows liposome stability (and permeability) to be regulated by perturbing the structural conformation of the liposome nanocarrier (stimuli-responsive liposome, SRL) [99]. In this case liposomes are able to react when activated by internal stimuli coming from target tissues (such as pH, ionic strength, redox potential, enzymes) or applied external stimuli coming from outside the organism (such as hyperthermia, ultrasound, and electro-magnetic fields). For example, pH-sensitive liposomes which are stable at the physiological pH of normal tissues, will be subjected to destabilization under acidic environment encountered generally in pathological tissues, thus facilitating the release of its active components nearby tumours or inflammation/infection regions, thus increasing the therapeutic efficacy [99].

An interesting class of triggerable liposomes are the thermosensitive liposomes (TSL), where the lipid structure can be modified by the presence of temperature-sensitive lipids [100,101,102]. Temperature-sensitive liposomes release their encapsulated drugs at the melting-phase transition temperature (*T_m_*), where the liposome permeability is influenced by the transition from the gel to the liquid crystalline phase of the lipid bilayer. For example temperature-sensitive liposomes often include dipalmitoylphosphatidylcholine as key lipid component, which presents a gel-to-liquid crystalline phase transition occurring at *T* = 41 °C. An example of a low temperature triggered TSL (LTSL), which has been developed by Celsion Corporation, is given by ThermoDox, a thermosensitive liposomes formulation containing Doxorubicin drug, and composed of liposomes 1,2-dipalmitoyl-sn-glycero-3-phosphocholine (DPPC): mono steroyl PC (MSPC): PEG2000-DSPE (86:10:4, molar ratio) [100]. ThermoDox in combination with hyperthermia (HT) treatment (involving the heating of tumours to temperature of up to 42 °C) has been tested in clinical trials against liver cancer [101]. A recent study evidenced that the use of high temperature triggered TSL (HTSL) formulation with less MSPC lipid content and a higher phase transition temperature of *T_m_* = 44 °C gives higher stability and less content leakage compared to conventional LTSL, thus suggesting that the *T_m_* of TSL should be increased to obtain improved drug delivery efficiency to tumour [102]. It is worth noticing that in most cases the employment of SRL raised critical issues concerning the potential toxicity of liposome synthetic components and therapeutic efficiency (e.g., difficulty of tissue location and remote triggering) [100].

### 10.3. Drug Delivery with Targeted Liposome (Active Targeting)

A promising approach to increase the specificity of drug delivery with minimal accumulation in non-target sites is obtained by conjugating, to the liposome surface, a specific ligand which is able to bind selectively to a given receptor on the target cell (active targeting) [99,103]. As previously stated, the ligand-receptor bindings are driven by reversible intermolecular forces, such as ionic and Van der Waals interactions or hydrogen bonds that ensure the reversibility of the association/dissociation of docking. A large variety of ligands (such as proteins, antibodies, peptides, lectins, aptamers, and vitamins) which can bind to overexpressed receptors on tumour tissues, have been investigated as biomarkers for targeted drug delivery [103]. Folic acid (FA), one of the most investigated targeting ligands, has a high binding affinity to folate receptors (FR), which are overexpressed in a wide range of FR-positive tumour types, including ovarian, lung, breast, brain, kidney, and colon cancers. Targeted liposomes, incorporating FA, tend to bind selectively to the FR and, by triggering cellular uptake via endocytosis, provide then a highly selective drug delivery strategy [104].

Recently, targeting a long-circulating PEGylated liposomal prodrug formulation of anti-tumour Mitomycin C to the folate receptor has been shown to enable a high liposomal drug payload and result in a stable formulation with reduced toxicity [105]. Moreover, stable Baicalin loaded folate-receptor-targeted liposomes showed higher cytotoxicity and cell uptake compared with non-targeted liposomes [106]. Interestingly, by introducing sliding tethered ligands with self-regulating length, additional topological interactions to the toolbox of ligand–receptor design have been recently demonstrated by Marques et al. [107], thus paving the way for the development of versatile and reusable bio-adhesive substrates for drug delivery and tissue engineering applications. Targeted liposomes have recently been the basic platform for the development of multifunctional prototypes containing diagnostic and therapeutic agents entrapped within the same theranostic nanocarriers [108]. For example, a new (theranostic) nanocarrier for tumour-specific imaging and therapy based on folate-conjugated liposome incorporating Ru(II) polypyridyl complexes was recently proposed [109]. In that case, the interaction between the vehicle and target HeLa cells (that overexpressed the folate receptor) brought to cell death by being exposed to laser irradiation contributed to the effective delivery of [Ru(phen)_2_(dppz)]^2+^ to the cytoplasm and subsequent photoinduced interaction. This approach demonstrates that prominent tumour selectivity and therapeutic ability can be achieved by the successful combination of tumour specificity of folate-conjugated liposomes with the imaging and therapeutic potential rendered by [Ru(phen)_2_(dppz)]^2+^. Although several innovative configurations for targeted delivery of drugs have been developed in nanomedicine at a preclinical level, up to now few have been admitted in clinical trials, while none of them have been approved for the market yet, as we will see later [110,111].

## 11. Market and Clinical Development of Liposome-Based Drugs Formulations

**Figure 10:**
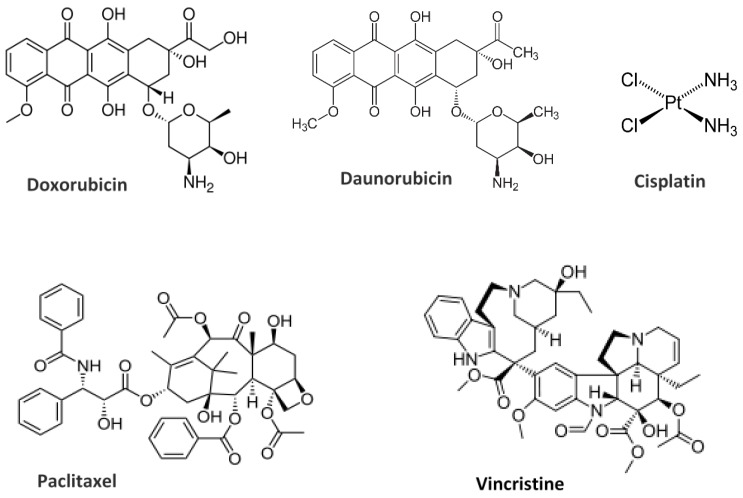
Chemical structures of drug molecules employed in liposomal formulations for clinical trials in the treatment of different typology of cancer.

Research and application of innovative solutions of liposome formulations, which have progressed toward a new generation of liposomes, are devoted mainly to cancer treatment and are mainly administrated intravenously, due to the high degradation of lipids in the gastrointestinal tract [110,111]. A special focus of the research is devoted to the investigation of the correlation of the blood circulation time and drug accumulation in target tissues with the specific physico-chemical properties of liposomal formulations investigated. Anti-cancer drugs Doxorubicin, Daunorubicin, Cisplatin, Paclitaxel, and Vincristine (Figure 10) are among the most extensively investigated agents for the liposome-based drugs formulations, and several liposomal formulations of these agents are currently in clinical use in cancer therapy.

Doxorubicin (or Anthracycline) is a potent chemotherapy drug largely employed in the treatment of a wide range of cancers, including blood malignant cancers (such as leukaemia and lymphoma), many types of solid (carcinoma), and soft (sarcomas) tissue tumours [110,111]. It slows (or stops) the growth of cancer cells by blocking isomerase 2, an enzyme that cancer cells need in order to divide and grow. In order to prolong the drug circulation time in the bloodstream and to avoid the MPS system, the doxorubicin is generally contained in PEGylated liposome nanocarriers. For example, a widely employed PEGylated liposomal doxorubicin formulation for the treatment of epithelial ovarian Kaposi's sarcoma is *Doxil*, composed of hydrogenated soy L-α-phosphatidylcholine (HSPC): cholesterol: PEG 2000-DSPE (56:39:5 molar ratio) and *LipoDox*, composed of 1,2-distearoyl-sn-glycero-3-phosphocholine (DSPC): cholesterol: PEG 2000-DSPE (56:39:5 molar ratio). A non-PEGylated version of liposomal doxorubicin formulation employed for treatment of recurrent breast cancer is provided by *Myocet*, composed of egg PC (EPC): cholesterol (55:45 molar ratio). Moreover, *DaunoXome* is a liposomal Daunorubicin formulation employed for the treatment of blood tumours, composed of DSPC and cholesterol (2:1 molar ratio). Finally, the *Marqibo* formulation, which is a liposome-based nanocarrier for the Vincristine chemotherapy drug, is employed for the treatment of metastatic malignant uveal melanoma. The previous five liposome-based drug formulations have been approved for intravenous application by the US Food and Drug Administration (FDA), and are already present in the market [110,111].

Other anti-cancer drugs formulated with liposomes are approved for (different stages) clinical studies. For example, a temperature-responsive version of PEGylated formulation of liposomal doxorubicin is provided by the nanocarrier Thermodox (composed of DPPC, mono steroyl PC (MSPC), and PEG2000-DSPE). Thermodox represents a triggerable release formulation, in phase III clinical trials, which is able to release its contents inside the tumour upon heat transfer, using a radio frequency ablation (RFA) process. 

Other important liposome nanocarriers include liposomal formulations for the *Paclitaxel* (or *Taxol*), a potent anti-cancer drug used to treat ovarian, breast, lung, and pancreatic cancers [110,111]. Two important Paclitaxel formulations, still in clinical trials, are *LEP-ETU* (composed of DOPC lipids, cholesterol, and cardiolipin) employed for treatment of ovarian, breast, and lung cancers and *EndoTAG-1* (composed of N-[1-(2,3-Dioleoyloxy)propyl]-N,N,N-trimethylammonium methyl-sulfate (DOTAP) and DOPC lipids) employed in anti-angiogenesis, breast, and pancreatic cancers treatments. Finally, *Lipoplatin* and *SPI-077*, which are liposomal formulations for the *Cisplatin* chemotherapy drugs, are in clinical trials for the treatment of head/neck and lung cancers [110,111].

## 12. Future Perspectives: The Theranostic Approach for Cancer Treatment

**Figure 11:**
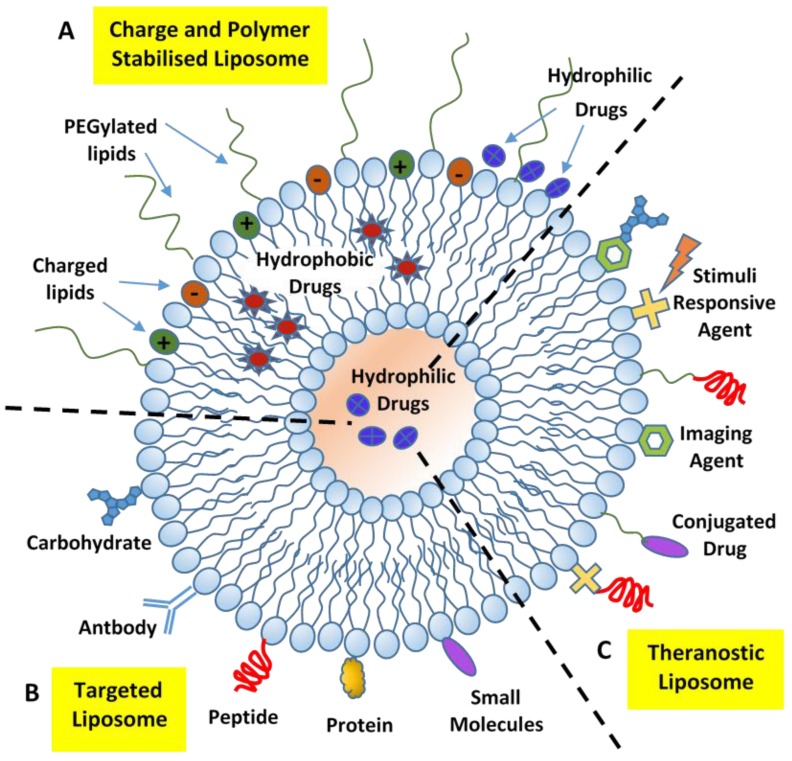
Schematic representation of the different types of liposomal drug delivery systems: Charge and polymer stabilized (**A**), targeted (**B**), and theranostic (**C**) liposomes.

Liposome-based nanocarriers have been the longest-studied platform and have attracted considerable attention for their extensive applications in biotechnology, drug, and gene delivery. Recent developments in nanoscience stimulated the combination of the simple approaches of liposome technology with the advanced concept of supramolecular self-assembly for the development of more complex, hierarchical nanostructures [108,112,113]. The introduction of multi-functional, multi-component formulations that perform (within the same liposome theranostic platform) different functions, such as the delivery of combinations of therapeutic drugs, site-specific targeting/imaging capabilities, and/or stimuli responsive assembly/disassembly processes to control drug release, allows for the development of a large variety of theranostic systems capable of performing a defined set of bio-medical tasks (Figure 11).

Owing to a facile modulation of their size, hydrophobic/hydrophilic character, low toxicity, and biocompatibility, liposomal nanocarriers still represent one of the best platforms that continues to attract the renewed interest of researchers who, through combining basic principles of soft-matter assembly with the more advanced concepts of supramolecular (bio-)chemistry, work towards designing and developing complex theranostic nanocarriers [108,114]. There is no doubt that, with additional understanding of the main soft interactions involved in basic processes of drug delivery, it will be possible to achieve a deeper insight into the structure-function relationship involved in relevant pharmacokinetic processes. Those circumstances will inspire the development of novel biomimetic strategies that can be translated into practical therapeutics for clinic and market development.

## 13. Conclusions

We presented recent advances in liposome based drug nanocarriers, by analyzing the relevant soft interactions involved in drug delivery processes which are responsible for the main behaviour of soft nanocarriers in complex physiological fluids. Relevant strategies to overcome the biological barriers during nanocarrier drug delivery are presented, outlining the main structure-properties relationship as well as their advantages (and drawbacks) in therapeutic and biomedical applications. The investigation of the soft interactions between liposomes at the molecular level can be considered to be an important framework for the modeling of the molecular recognition processes occurring between cells. These relatively weak interactions can be amplified by multiple binding at the liposome surface in multivalent complexes. While considerable research effort on the theoretical side is certainly necessary, further investigations of physico-chemical properties are required with different/complementary experimental methods in order to critically evaluate the stability of the nanocarriers in simulated environmental solutions which are close to the physiological fluid conditions.

## Figures and Tables

**Table 1:** Strength and characteristic ranges of the main soft interactions.

Interaction Type	kJ/mol	Distance range (nm)
Covalent bond	100–400	0.07–0.50
Hydrogen bond	4–120	0.3
Hydrophobic interaction	<40	variable
Electrostatic/ionic interaction	20	0.25
Van-der-Waals interaction	0.4–5	0.3–0.6
Steric stabilization	variable	variable
